# Assessment of population genetic structure in the arbovirus vector midge*, Culicoides brevitarsis* (Diptera: Ceratopogonidae), using multi-locus DNA microsatellites

**DOI:** 10.1186/s13567-015-0250-8

**Published:** 2015-09-25

**Authors:** Maria G Onyango, Nigel W Beebe, David Gopurenko, Glenn Bellis, Adrian Nicholas, Moses Ogugo, Appolinaire Djikeng, Steve Kemp, Peter J Walker, Jean-Bernard Duchemin

**Affiliations:** CSIRO Health & Biosecurity Australian Animal Health Laboratory, 5 Portalington Road, Geelong, Victoria 3220 Australia; School of Medicine, Deakin University, 75 Pidgons Road, Waurn Ponds, Victoria 3216 Australia; School of Biological Sciences, The University of Queensland, St Lucia, Queensland 4072 Australia; CSIRO Health & Biosecurity Ecosciences Precinct, 41, Boggo Road, Dutton Park, Queensland 4102 Australia; NSW Department of Primary Industries, Wagga Wagga Agricultural Institute, PMB, Wagga Wagga, New South Wales 2650 Australia; Graham Centre for Agricultural Innovation, Locked Bag 588, Wagga Wagga, New South Wales 2678 Australia; Northern Australia Quarantine Strategy, 1 Pederson Road, Marrara, Northern Territory 0812 Australia; International Livestock Research Institute, P.O. Box 30709, 00100 Nairobi, Kenya; Biosciences eastern and central Africa – ILRI Hub (BecA-ILRI Hub), ILRI, PO Box 30709, 00100 Nairobi, Kenya

## Abstract

Bluetongue virus (BTV) is a major pathogen of ruminants that is transmitted by biting midges (*Culicoides* spp.). Australian BTV serotypes have origins in Asia and are distributed across the continent into two distinct episystems, one in the north and another in the east. *Culicoides brevitarsis* is the major vector of BTV in Australia and is distributed across the entire geographic range of the virus. Here, we describe the isolation and use of DNA microsatellites and gauge their ability to determine population genetic connectivity of *C. brevitarsis* within Australia and with countries to the north. Eleven DNA microsatellite markers were isolated using a novel genomic enrichment method and identified as useful for genetic analyses of sampled populations in Australia, northern Papua New Guinea (PNG) and Timor-Leste. Significant (*P* < 0.05) population genetic subdivision was observed between all paired regions, though the highest levels of genetic sub-division involved pair-wise tests with PNG (PNG vs. Australia (F_ST_ = 0.120) and PNG vs. Timor-Leste (F_ST_ = 0.095)). Analysis of multi-locus allelic distributions using STRUCTURE identified a most probable two-cluster population model, which separated PNG specimens from a cluster containing specimens from Timor-Leste and Australia. The source of incursions of this species in Australia is more likely to be Timor-Leste than PNG. Future incursions of BTV positive *C. brevitarsis* into Australia may be genetically identified to their source populations using these microsatellite loci. The vector’s panmictic genetic structure within Australia cannot explain the differential geographic distribution of BTV serotypes.

**Electronic supplementary material:**

The online version of this article (doi:10.1186/s13567-015-0250-8) contains supplementary material, which is available to authorized users.

## Introduction

Bluetongue (BT) is an economically important viral disease throughout tropical and temperate regions of the world, posing a threat to the livestock industries, through production losses and negative impacts on trade [1]. The disease affects primarily sheep and goats. Cattle can also be infected but rarely show signs of disease [2]. Biting midges (*Culicoides* spp.) are vectors of bluetongue virus (BTV). In Australia, *C. actoni* Smith, *C. brevitarsis* Kieffer, *C. fulvus* Sen and Das Gupta are proven vectors of BTV and several others including *C. brevipalpis* Delfinado, *C. dumdumi* Sen and Das Gupta *C. oxystoma* Kieffer, *C. peregrinus* Kieffer and *C. wadai* Kitaoka are regarded as potential vectors [3,4]. Of these species, *C. brevitarsis* is the most widely distributed throughout northern and eastern parts of the continent [5,6], and is considered to be the major vector, employing cattle and buffalo dung as breeding sites [4,7].

BTV appears to have been introduced to Australia from Southeast Asia on multiple occasions by infected wind-borne vectors [8,9]. Indeed, 10 of the 26 known BTV serotypes have been detected in Australia through intensive surveillance during the past 30 years and there is evidence that at least four of these serotypes were introduced since the surveillance programme commenced [10]. The absence of clinical bluetongue disease in Australia, despite evidence of widespread infection in cattle, has been attributed to the limited distribution of *C. brevitarsis* to non-sheep breeding regions, primarily in the south of the continent and the relatively low pathogenicity of Australian BTV serotypes. Surveillance has indicated that the distribution of BTV serotypes in Australia is asymmetric with all 10 serotypes detected in the far northern region and only two serotypes (BTV-1 and BTV-21) enzootic in the southern portions of the eastern states. The factors influencing the distribution of serotypes are unknown and there is concern that introductions of exotic BTV strains from Southeast Asia via windborne *Culicoides* could destabilize the current situation [9]. A recent study of long-distance dispersal of *Culicoides* midges using an aerial migration model, indicated that migration of *Culicoides* into northern Australia from Timor-Leste (TL) and Papua New Guinea (PNG) is possible with Timor considered the most likely source of incursions [11]. Recent phylogeographic analyses [12,13] generally support those contentions and further indicate *C. brevitarsis* likely entered Australia and PNG separately from independent southeast Asian sources, in recent historical times [13]. Results of those prior genetic studies were based on analyses of a single maternally inherited gene and are potentially biased by a variety of evolutionary, demographic and sampling processes [14,15]. Additional population genetic analyses using multiple independent loci are needed to test hypotheses concerning the origins of recent arrivals of midge species in Australia.

The first aim of this study was to develop a technical workflow for identifying DNA microsatellite markers *de novo* from small organisms such as *Culicoides* from which limited quantities of genomic DNA can be extracted. The second aim was to identify and compare allelic diversity of microsatellite loci among *C. brevitarsis* in Australia and neighbouring countries (PNG and TL) that are suspected sources of *Culicoides spp*. entering Australia. In this latter aim, we also sought to determine the levels of population genetic connectivity among the regions and infer the likely source(s) of midges in Australia during historical and current times.

## Materials and methods

### Insect sampling and DNA preparation

**Figure 1:**
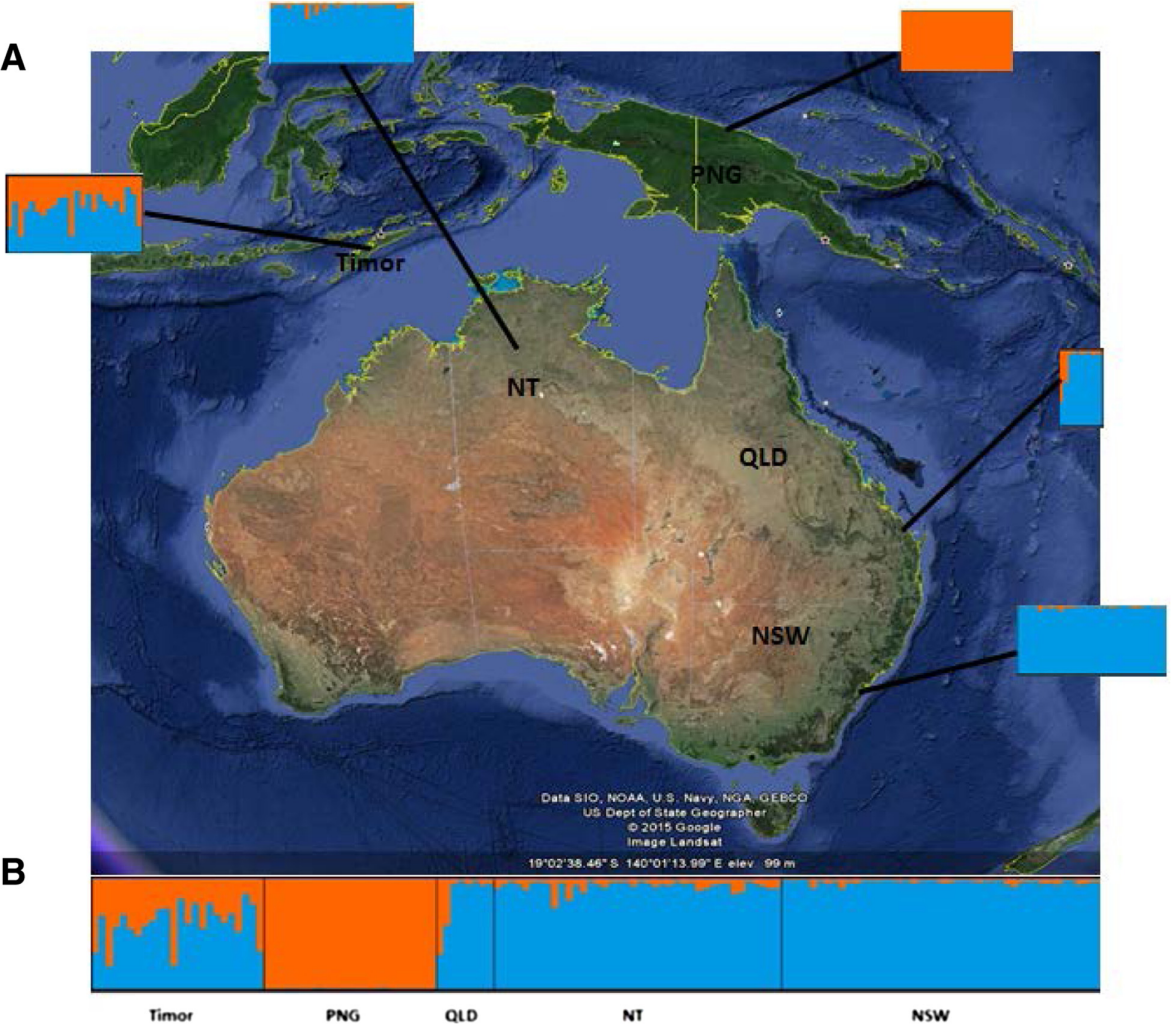
**Sites of collections of** ***C. brevitarsis*** **and plot of the genetic structure in this study.** **A** STRUCTURE plot results (K = 2) integrated into a map showing the locations of the study area. **B** The Q matrix derived from STRUCTURE clustering analysis show the inferred ancestry membership proportions of each individual in each cluster (K = 2). Each individual is represented by a single vertical line, partitioned into K colored segments that represent the individual’s estimated membership fraction in each of the K inferred clusters. The X-axis corresponds to the pre-defined populations (TL, PNG, QLD, NT and NSW) and the Y-axis represents the proportional estimates of the estimated membership in clusters, which add up to one.

A total of 141 samples were collected using light trap or sweep net, preserved in 70% ethanol and identified from sites in the Northern Territory (NT), Queensland (QLD), New South Wales (NSW), PNG and TL (Figure 1A) as described by Gopurenko et al. [13]. Species identification was verified using genetic methods reported in [16,17] to ensure morphologically related species of *Culicoides* were not included in analyses. After species identification, total genomic DNA was extracted from single specimens of *C. brevitarsis* using the DNeasy blood and tissue kit (Qiagen) according to the manufacturer’s protocol and by a non-destructive genomic DNA extraction method [16]. The genomic DNA was quantified using a Qubit fluorometer (Life Technologies, Invitrogen).

### Whole genome amplification of *C. brevitarsis*

To improve the amount of genomic DNA yield from singleton *Culicoides* for downstream manipulations, multiple displacement amplification-based (MDA) whole genome amplification (WGA) using Repli-g ultrafast mini kit (QIAGEN) was conducted on specimen DNA according to the manufacturers’ protocol. To assess the differences in yield of amplification from a range of starting amounts of genomic DNA and according to two different denaturing procedures, 10.7 ng and 0.215 ng of DNA were denatured either by heat (95 °C for 3 min) and or by adding denaturing solution (buffer D1; QIAGEN) and then amplified using the REPLI-g ultrafast mini kit at 30 °C for 1.5 h followed by polymerase inactivation at 65 °C for 3 min. Water was used as a negative control. The resulting DNA was quantified using a Qubit fluorometer and qualitatively assessed by electrophoresis (1% agarose gel (Invitrogen) at 7.40 V/cm) in the presence of 1 kb DNA ladder and staining/UV visualisation.

### Quality check of whole genome amplified DNA

To evaluate the quality of the whole genome amplified DNA, the products of the previous amplification (together with the negative control) were used as templates to PCR amplify a 600 bp housekeeper gene (actin). Each 20 μL reaction included 1 μL template DNA (approximately 20 ng), BSA (400 ng/μL), 5 μm each forward and reverse primer (Act-2 F and Act-8R) [18] and 15 μL Platinum PCR supermix high fidelity (Life Technologies). The cycling profile was 94 °C for 2 min, 35 cycles of 94 °C for 30 s, 58 °C for 30 s, 72 °C for 30 s and a final elongation at 72 °C for 5 min [18]. The PCR products were assessed for size by electrophoresis as described earlier.

### Isolation and screening of microsatellite repeats and primer design

To isolate DNA microsatellite markers, 1 μL of a pooled specimen DNA (*n* = 7 New South Wales, *n* = 8 Northern Territory) was whole genome-amplified. The amplified whole genomic DNA was sequenced on 1/8 plate Roche 454 Genome Sequencer FLX plus by an external contractor (Macrogen, Geumchun-gu, Seoul). The raw sequence reads of amplified whole genomic DNA were screened directly for di-, tri- and tetranucleotides using MSATCOMMANDER v0.8.2 [19]. Primers were designed to the flanking regions of the microsatellite repeats using the Primer 3 program [20]. The primers were checked for similarities amongst each other and were blasted against the NCBI database using BLASTN to determine if the target sequences were derived from microorganisms and or other contaminants. Primers that were found to be unique i.e. neither homologous to sequences from other organisms in the database nor similar to each other were subsequently compared to our sequenced *C. brevitarsis* raw reads. The primer pairs that were predicted to anneal to more than one read were aligned to those reads after reverse-complementing the reverse primer.

### Microsatellite validation

Initially, 38 primer pairs flanking dinucleotide repeats were selected for validation. Each primer pair was tested on 10 individuals. Twelve of the primer pairs that amplified 100% of the sub-set of samples (Table 1) were used to amplify DNA from 141 individuals (one individual acting as the positive control and reference allele size) sampled from three regions in Australia (Northern Territory; southeast Queensland and New South Wales) as well as from northern Papua New Guinea and Timor-Leste (Figure 1A). Each 25 μL PCR reaction included 1.0 μL template DNA (20 ng), 0.2 μmol (each) forward and reverse fluorescently labeled primer (Applied Biosystems, USA), 18 μL (0.396 U) of Platinum PCR supermix high fidelity (Life Technologies) and 2.75 μL de-ionized water (Life Technologies). The amplification was carried out under the following conditions: initial denaturation of 94 °C for 3 min, then 15 cycles of 94 °C for 30 s, 60 °C for 30 s with a gradient decrease of 1 °C/cycle, 72 °C for 30 s followed by 30 cycles of 94 °C for 30 s, 45 °C for 30 s, 72 °C for 30 s and a final elongation step of 72 °C for 7 min. Amplified PCR products were fragment-sized by an external contractor (Macrogen, Geumchun-gu, Seoul). The fragment lengths were analyzed and corrected manually using Genemapper v4.0 (Applied Biosystems). A fraction of the original sample size (6% of specimens) was re-run using touchdown PCR conditions (annealing temperature reduced to 40 °C) as a validation of initial allelic scores.

**Table 1:** **Microsatellite loci and primers developed in this study.** Locus G7B17 excluded from population genetic due to evidence of significant (*P* < 0.05) linkage to locus GO2AH

Locus	Motif type	Range of sizes	N_A_	Left primer	Right primer	#PROBEDB_ACC
HH82P	(AC)^13	400–422	13	CACCTCTGAGAAATCCAACCG	AGTTGGTCAGCACCTCAAG	Pr032367671
GU21Z	(GT)^14	210–222	6	TGAGTTCGTATGGCAAGGC	ACAGCGAAATGTTCATACGTG	Pr032367668
G7B17	(CA)^11	204–208	3	ATGGGCGAACAAATCGAGG	AACATTCGTCTTCGCTGCC	Pr032367664
HIIUN	(GT)^12	314–328	8	ATCCGGGAATACCTGCGAG	AAGTGTTGCCGTCGATTTC	Pr032367672
HNBZE	(CA)^9	328–344	9	GTGTCCGTAGCGAGTAGCC	AGCACGATTGAAACCGACAG	Pr032367673
G9WRZ	(GT)^8	400–412	6	GCTACTGGAGCGATCTAACG	ATTAGTGTGCCGCCTTCAG	Pr032367665
G5L7G	(AC)^9	398–412	8	AGCATGATGAAATGTCCCGC	TCAACTACTGCTGCCCGAG	Pr032367663
GO2AH	(GT)^8	178–194	8	TGGCTGCGAGTCGAGATG	GCCGTCGATAAGAATTAAGGTAAAC	Pr032367666
GONNE	(CGG)^8	304–316	4	TGATGCCCGTCCAAGATCC	GTTGCTCCGTAGTCGAACG	Pr032367667
G1FMO	(AC)^11	300–322	7	GCGTCATCAGTGCCAAGAC	GGAACTACACGGAGCAAGC	Pr032367662
HBCQD	(GT)^10	368–386	9	GCATTTGCGTTTGGCGATG	GAAGGCGTCATTCGATTTGC	Pr032367670
GU6HJ	(AG)^8	190–194	3	GGCGATGACGATAACGAGC	ACATGACTTTGAAATTGAATCTGCC	Pr032367669

### Data analysis

Genepop [21] was used to test for linkage disequilibrium between each pair of loci and across regions (Fischer’s method) and to estimate allelic diversity and the coefficient of inbreeding (F_IS_) at individual loci within populations. Microchecker [22] was used to check for putative null alleles, large allele dropout or stutter peaks.

Deviations from the Hardy-Weinberg equilibrium was estimated by using Arlequin v3.0 [23]. The observed numbers of heterozygotes and homozygotes at loci in each population were tested against the expected numbers using a chi-square test.

To infer population structure from the microsatellite data, multilocus genetic distance [24] and fixation index (F_st_) [25] estimates were calculated between population pairs using Genepop, Arlequin and GenAlex v6.5 [21,23,26]. Permutation tests (100 replications) were used to determine significance of the population structure estimates.

A Bayesian clustering approach implemented in STRUCTURE v2.3.4 [27] was used to provide probabilistic estimates of population structure based on unlinked multi-locus genotype distributions. Individuals are assigned probabilistically to (K, where K may be unknown) populations or jointly to two or more populations if their genotypes indicate they are admixed. The model doesn’t assume a particular mutation process [27].

To generate a matrix of individual membership co-efficient and population ancestry components, the following parameter set was applied: burnin period of 100 000, Markov chain Monte Carlo (MCMC) repeats of 100 000, ancestry model of admixture of LOCPRIOR model and a K value range from 1–10 with an iteration of 22.

STRUCTURE HARVESTER v0.6.94 [28] was used to infer the most likely number of genetic clusters (K) present using both the Evanno and Delta K methods while CLUMPAK [29] was used to collate the data into a single matrix for all the K values.

## Results

### WGA of heat and D1 buffer-denatured DNA and quality assessment of whole genome amplified DNA

The DNA yield from WGA DNA of both heat-denatured and denaturing solution (buffer D1) denatured DNA was compared. The D1 buffer-denatured DNA yielded more WGA product than the heat-denatured DNA (data not shown). This suggests the superiority of the D1 buffer over heat as a denaturing agent in WGA. Tests using low concentration DNA sample template (0.215 ng) resulted in higher yield of WGA products compared to yield using higher concentration DNA template (10.7 ng). The size of WGA DNA smears was from >10 kb with a smear extended down to ~1 kb. The negative control reaction did not amplify. Positive controls based on actin gene amplification were successful in both the whole genome amplified heat and D1 buffer-denatured DNA. The negative control reaction from the same WGA reaction did not amplify.

### Isolation and screening of microsatellite repeats and primer design

**Figure 2:**
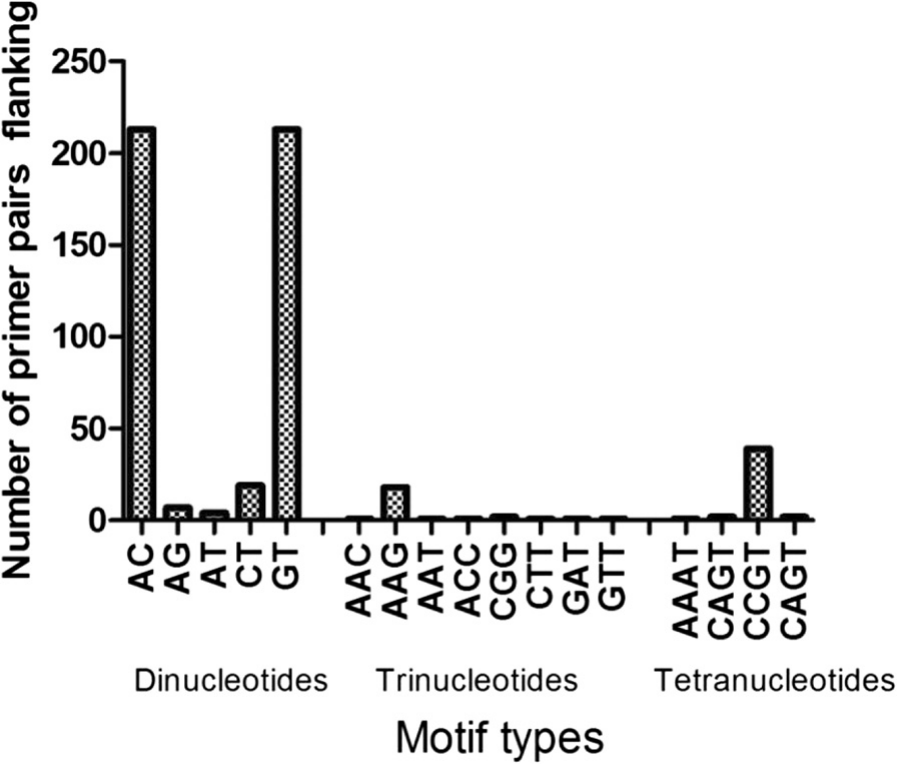
**The distribution pattern of primers across the different microsatellite repeats in** ***Culicoides brevitarsis*** **genome.** Considerable variation was observed among the 12 validated microsatellite loci. Exact tests for linkage disequilibrium identified significant association between locus G7B17 and GO2AH. Subsequently locus G7B17, which was less polymorphic than GO2AH was excluded from downstream analysis. The number of alleles per locus ranged from 3–13 and 78 alleles were scored across the 11 loci.

A total of 120 005 reads of 414 average read length was obtained after sequencing the amplified whole genomic DNA. The maximum theoretical genome coverage obtained in this study was ~0.25 X (no. of reads X average read length/size of genome) of the approximately 200 MB *Culicoides* genome [30]. A total of 2091 reads were found to contain putative microsatellite repeats. From the reads that contained putative repeats, 2594 putative microsatellite repeats were isolated (2361 dinucleotide repeats, 90 trinucleotide repeats and 98 tetranucleotides repeats). We detected approximately 52 microsatellite repeats per MB of the genome with most primers flanking AC and GT repeats (Figure 2).

A total of 526 primers were designed to the flanking regions of the microsatellite repeats. From these, 420 primers were either found to be similar to each other or homologous to sequences from other organisms in the database, while 106 primers were unique and were not homologous to anything in the NCBI database. After comparing the 106 unique primers against the sequenced reads, 93 primer pairs (Additional file 1) were predicted to anneal to a single unique read while 13 primer pairs were predicted to anneal to more than one read or to more than one site on a single read. High conservation between the primer pairs and the reads was evident upon aligning them to the reads. These particular primer pairs were excluded from the study to avoid multi copy amplification that may complicate the analysis procedures downstream. A subset of 38 primers was used to amplify ten individuals. Twelve of these primer pairs PCR amplified in 100% of the individuals. The remainders amplified inconsistently and were not used further (Additional file 2).

### Allele frequencies distribution, heterozygosity, Hardy-Weinberg equilibrium and linkage disequilibrium

Considerable variation was observed among the 12 validated microsatellite loci (Table 1 and Additional file 3). Exact tests for linkage disequilibrium identified significant association between locus G7B17 and GO2AH. Subsequently locus G7B17, which was less polymorphic than GO2AH (Table 1) was excluded from downstream analysis. A summary of genetic diversity within the five regional populations was calculated for the remaining 11 loci (Additional file 3). The number of alleles per locus ranged from 3–13 (Table 1) and 78 alleles were scored across the 11 loci. Expected gene diversity (H_e_) varied from 0.01–0.89 and in 96% of comparisons was higher than the observed heterozygosity (H_o_) with a range of 0–0.88. The inbreeding coefficient (F_IS_) was significantly different from zero in an average of eight loci per population. Estimates of F_IS_ differed between populations such that NT and NSW had a higher frequency of loci showing significant inbreeding compared to the other populations. Locus HBCQD was monomorphic in TL, PNG and QLD but polymorphic in NT and NSW. Global tests by locus revealed departure from Hardy-Weinberg equilibrium for most loci among all the populations. While putative null alleles were identified at some loci in some populations using Microchecker, the null alleles were not locus specific (Additional file 3) and a large proportion (73%) of data of repeated PCRs were congruent with the initial results.

### Population genetic structure

Statistically significant genetic differentiation was demonstrated between the northern PNG population and the other populations in three tests employed. Both the Nei genetic distance and F_ST_ estimates indicated presence of population structure among all three regions. Nei’s genetic distance varied from 0.03–0.31 while the F_ST_ estimates varied from 0.01–0.19 (Table 2). Permutation tests in all cases indicated that genetic structure between paired regions was significant (*P* < 0.05). Paired region F_ST_ estimates indicated the highest levels of sub-division between PNG and Australia (F_ST_ = 0.12) and between PNG and Timor-Leste (F_ST_ = 0.095). In contrast, F_ST_ between Australia and Timor-Leste (0.03) was lower (Table 3). There was no evidence of any significant population structure between Australian populations (Table 2).

**Table 2:** **Estimated genetic distance**

	TL	PNG	QLD	NT	NSW
TL		0.17	0.14	0.1	0.09
PNG	0.09(0.0 + - 0.0)		0.31	0.27	0.26
QLD	0.08(0.0 + - 0.0)	0.19(0.0 + - 0.0)		0.1	0.08
NT	0.04(0.0 + - 0.0)	0.12(0.0 + - 0.0)	0.06(0.0 + - 0.0)		0.03
NSW	0.03(0.0 + - 0.0)	0.13(0.0 + - 0.0)	0.04(0.06 + -0.02)	0.01(0.08 + -0.03)	

Nei genetic distance values (above diagonal) and pair-wise fixation index (F_ST_) values (below diagonal) for sampled Australasian populations of *C. brevitarsis.*

Significance level = 0.05.

**Table 3:** **Estimated genetic distance**

Timor	PNG	AUS	
	0.177	0.082	Timor
0.10 (0.0 + -0.0)		0.251	PNG
0.03 (0.0 + -0.0)	0.12(0.0 + -0.0)		AUS

Nei genetic distance values (above diagonal) and pair-wise fixation index (F_ST_) values (below diagonal) for sampled regional populations of *C. brevitarsis.*

Significance level = 0.05.

Evanno and Delta methods identified a most probable clustering value of K = 2, for defining population group structure analysed under STRUCTURE. The clustering analysis (Figure 1B) identified inferred ancestry membership proportional probabilities of each specimen, in the optimal two cluster arrangement (Additional file 4). The posterior probability values indicate individuals from regions in Australia (NT, NSW and QLD) are of admixed ancestry with the largest proportion of their ancestry assigned to cluster 2. Timor-Leste population ancestry on the other hand is derived almost equally between cluster 1 and 2. The PNG population is almost exclusively assigned to cluster 1.

## Discussion

Microsatellite loci are regarded as powerful molecular markers because of their high mutability, co-dominance, abundance in the genome and relative ease of scoring [31,32] and have been employed to study vectors of medical and economic importance [33–37]. However, isolation of microsatellite markers from tiny and non-cultivable organisms has been hampered by the limited quantity of available genomic DNA. Recently, a study by Mardulyn et al. [37] successfully isolated 10 microsatellite markers from *C. imicola* (1–5 mm) using recombinant approaches as described by Glenn et al. [38] and utilized the microsatellites to better understand the mechanism responsible for the northward spread of bluetongue in the Mediterranean region. In contrast, the present study isolated microsatellite markers from *C. brevitarsis* by initially increasing the amount of genomic DNA by whole genome amplification (MDA technique) and subsequently sequencing the whole genome amplified DNA on an eighth of a lane of a 454 GS FLX sequencer. To the best of our knowledge, this is the first study that has isolated microsatellite markers *de novo* from WGA DNA for any organism. The MDA-based whole genome amplification procedure provides an ideal method of isolating microsatellite markers from limited amounts of genomic DNA. This gave rise to 120 005 raw reads from which 93 primer pairs that are unique to a single read (and not the products of contamination) were isolated. The abundance of microsatellite repeats may be skewed due to a potential unbalanced representation of the genome during whole genome sequencing. A selected number of markers flanking the repeats were successfully validated and utilized to study Australasian populations of *C. brevitarsis.*

Aerial arthropod dispersal modeling studies have assessed the possible routes of introduction of BTV vectors into Australia from neighboring countries including Timor-Leste and PNG. The models predict stronger pathways of dispersal from Timor-Leste to Australia, than from PNG [8,11,39]. Mitochondrial DNA (mtDNA) evidence indicates several Southeast Asian *Culicoides* species have entered northern Australia from both Timor-Leste and PNG [12]. More recent mtDNA evidence indicates historical pathways of dispersal by *C. brevitarsis* to northern Australia are less certain. Based on their study of COI haplotypes, Gopurenko et al. [13] found evidence of multiple independent range expansions of the species into Australasia, with evidence of separate expansions of the species into north Australia and PNG from independent Southeast Asia sources. They also identified moderate levels of gene flow in directions contrary to expectations of the historical dispersal of the species based on growth of the cattle industry in Australasia. The authors concede that gene flow estimates in their study were historical and likely influenced by migrations to the region from unsampled Southeast Asian sources.

So far, no genetic studies have used multi locus markers to examine population structure of this species. The current study applied 11 newly isolated microsatellite markers to determine the population genetic structure of *C. brevitarsis* in Timor-Leste, Australia and northern PNG.

The results here indicate populations of *C. brevitarsis* in Australia are more similar to those in Timor-Leste than in northern PNG. This is particularly evident in STRUCTURE analyses, which have assigned Australia and Timor-Leste specimens to a population cluster separate from a single PNG cluster. Pairwise F_ST_ tests indicate significant structure exist between Australia and its northern neighbors, but the level of F_ST_ structure evidenced between these two regions is less than that in pair-wise comparisons with PNG. The results demonstrate that *C. brevitarsis* in Australia was potentially introduced into NT from Timor-Leste or from neighboring islands in Indonesia (less likely from PNG) consistent with previous spatial modeling studies that indicated the islands of Timor and Sumba in the Indonesian Archipelago were the likely principal sources of *Culicoides* dispersal into northern Australia [8,11,39]. The gene flow barriers between northern PNG and other Australasian populations would mainly be geographic. High mountain ridges characterize PNG spatial geographical features with sharp narrow crests separated by deeply incised V-shaped valleys. The main geographical barrier to gene flow from northern PNG to Australia would be the Central and Owen Stanley Range of PNG highlands that span a distance of 200 km from the central cordillera and with an altitude of 4000 m above sea level [40]. The high peaks would result in a rough terrain that has been shown to act as an airflow barrier, which would slow dispersal of *Culicoides* on land [41]. In contrast, the relatively low plateau geography of Australia is likely to have given rise to the genetically homogenous population across the continent [10].

Genetic sampling of *C. brevitarsis* from northeastern Australia and southern PNG was not conducted in this study and future genetic work is required to examine levels of population connectivity between the two regions and to test the possibility of separate entry of BTV infected midges into northeastern Australia from the southern PNG region. The movement of other species of *Culicoides* from southern PNG into northern QLD has been documented [12] so movement of *C. brevitarsis* appears likely. Our starting working hypothesis was to highlight the potential larger genetic difference between the most distant NT and NSW regions and we placed more emphasis in the sampling of these two regions rather than the intermediate Queensland area. This has lead to a weaker significance of these last samples over the rest of the individuals.

Furthermore, both STRUCTURE analysis and conventional F_ST_ estimates of population subdivision indicate *C. brevitarsis* populations are genetically panmictic in Australia and display no evidence of genetic separation or structure between northern and eastern sampled populations. This result suggests that the presence of the two BTV episystems in Australia (northern and eastern) as demonstrated by the distribution of BTV serotypes is not as a result of genetic structuring of this vector and is due to other factors for example, the presence of other vector species in northern Australia that are absent from southeastern Australia.

The 11 loci isolated and validated in this study were polymorphic with number of alleles ranging from 3 to 13. Departure from Hardy-Weinberg equilibrium resulting from excessive homozygote presence in one or more sampled populations was evident at all 11 loci. Severe inbreeding can cause an excess of homozygotes across loci, however, this would involve substantial and or ongoing bottlenecking of populations down to few breeding adults [42]. Substantial population bottlenecks would however have greater and immediate effect on haploid genetic markers such as maternally inherited mtDNA [43]. MtDNA evidence obtained by Gopurenko et al. [13] indicated moderate to high levels of mtDNA diversity among *C. brevitarsis* populations in the same regions examined in the present study and further their modeled estimates of effective maternal population size in contemporary populations was in the order of hundreds of thousands of individuals. Alternatively, null allele presence, caused by mutations at locus specific primer annealing sites which retard PCR amplification efficiency of particular alleles, can result in excesses of homozygotes scored at loci and hence significant deviation from Hardy-Weinberg Equilibrium in populations [44]. Allelic dropout in PCR caused by a variety of inhibitory causes can result in similar outcomes [45].

To validate the levels of potential allelic and genotypic miss-scores caused by a variety of PCR processes including PCR dropout and primer redundancy, a total of 86 DNA samples initially identified either as homozygous at one or more loci or had failed to amplify were re-amplified at one or more of the 11 markers using a touchdown PCR program. Three percent of samples initially identified as homozygous were scored as heterozygotes in the repeat PCRs, 17% of samples that failed to amplify previously were successfully re-amplified, 7% of samples that had been amplified before failed to amplify during this re-amplification experiment and 73% were unchanged.

The effects of null allele on test for HWE have been reported earlier [46]. The effects of null allele on genetic test of population structure vary according to the severity of the null allele presence and the type of test being used. Carlsson [47] examined effects of null allele on population structure estimated by F_ST_ and also population assignment testing (as employed in STRUCTURE) and they identified significant but small upwards biases to F_ST_ (F_ST_ increased between 0.003 and 0.004) and slight reduction in the power of STRUCTURE to correctly assign individuals to populations (0.2 and 1.0% units). With these caveats, we argue the likely slight upward biases to our pairwise estimates of F_ST_ as well as some effects on specimen cluster assignment using STRUCTURE, which would not drastically change the results of our study.

We have demonstrated that whole genome amplification of the genomic DNA and subsequent whole genome sequencing resulted in a successful *de novo* isolation of microsatellite markers from *C. brevitarsis*. These microsatellite markers are likely to be very useful for genetically typing population origins of *C. brevitarsis* detected in the future. They can also be applied to carry out a broader analysis of gene flow in Australasian and Southeast Asian populations of *C. brevitarsis*.

## Additional files

Additional file 1:
**A summary file of the total number of primers designed to the flanking regions of the microsatellite repeats.** From the total primers isolated, 420 primers were either found to be similar to each other or blasted to sequences from other organisms in the database, while 106 primers were unique and did not blast to anything in the NCBI database. After blasting the 106 unique primers against the sequenced reads, 93 primer pairs were predicted to anneal to a single unique read while 13 primer pairs were predicted to anneal to more than one read or to more than one site on a single read. (XLS 36 kb) Additional file 2:
***C. brevitarsis*** **primer optimization.** A subset of 38 primers was used to amplify 10 individuals. Twelve of these primer pairs PCR amplified 100% of the individuals. The remainders amplified inconsistently and were not used further. (XLS 28 kb) Additional file 3:
**Summary of allelic variation in 11 microsatellite loci.** Allelic variation among *N* = 141 [Timor-Leste (*N* = 24), Papua New Guinea (*N* = 24), Queensland (*N* = 8), Northern Territory (*N* = 40) and New South Wales (*N* = 45)] *C. brevitarsis* specimens sampled from regional populations. N_allele_- Null allele, ^**m**^ - monomorphic locus, observed (H_o_) and expected (H_e_) heterozygosity, probability of deviation from Hardy-Weinberg Equilibrium (P_HWE_) and inbreeding co-efficient (F_IS_) as described. Significantly deviated F_IS_ values (*P* < 0.05) highlighted in BOLD type. (XLS 38 kb) Additional file 4:
**Probability values of population assignments for samples K = 2.** The population Q matrix derived from STRUCTURE clustering analysis show the inferred ancestry membership proportions of each individual, in each cluster. Each individual is represented by a single vertical line, partitioned into K colored segments that represents that individual’s estimated membership fraction in each of the K inferred clusters. The X axis corresponds to the pre-defined populations (TL, PNG, QLD, NT and NSW) and the Y axis represents the proportional estimates of the estimated membership in clusters which add up to one. (TXT 11 kb)
